# Women with premenstrual syndrome exhibit high interoceptive accuracy, but low awareness, with parasympathetic rebound responses from stress

**DOI:** 10.3389/fnins.2025.1489225

**Published:** 2025-02-17

**Authors:** Yumiko Crysia Suzuki, Hideki Ohira

**Affiliations:** Department of Cognitive and Psychological Sciences, Graduate School of Informatics, Nagoya University, Nagoya, Japan

**Keywords:** premenstrual syndrome (PMS), interoceptive accuracy, interoceptive awareness, emotion, stress, interoception and mental health, autonomic nervous system, premenstrual symptom

## Abstract

Premenstrual syndrome (PMS) is characterized by emotional or physical discomfort that occurs specifically during the luteal phase. However, women with PMS typically do not exhibit abnormalities in serum sex hormone levels or structural anomalies, making it challenging to identify distinct pathological findings unique to PMS. Instead, they may exhibit hypersensitivity to fluctuations in sex hormone levels within the normal range. This study involved experiments conducted in the late luteal phase. The pre-stress baseline state evaluated the effects of PMS on interoception using the Heartbeat Counting Task (HCT) and the Multidimensional Assessment of Interoceptive Awareness (MAIA), and the effects on emotional states using a questionnaire assessing subjective emotions in the late luteal phase. The results indicated that the “PMS group” scored higher on the HCT compared to the “without PMS group,” while their MAIA scores were lower. Additionally, the PMS group reported experiencing more negative and stressful states. The findings suggest that women with PMS demonstrate high accuracy in but lower awareness of interoception. This mismatch between “accuracy” and “awareness” may indicate a maladaptive state regarding mental health. The stress-related analysis examined whether the effects of stress on exacerbating PMS include impacts on interoception and the autonomic nervous system (ANS). States before baseline, immediately after, and during stress recovery were evaluated. The results revealed that women in the PMS group exhibited more pronounced negative and stressful states; they showed higher scores on the HCT, with scores progressively increasing as the task was repeated. Regarding ANS indices, no main effect of group was observed, but parasympathetic activity increased during the recovery period compared to the pre-stress baseline. In the degree of changes in parasympathetic activity from the baseline to post-stress and recovery periods, a group-by-time interaction effect was observed. These findings suggest that the PMS group may experience a prolonged rebound effect during the recovery phase following induced stress. In conclusion, women with PMS exhibit a discrepancy between high interoceptive accuracy and low awareness of their sensations. This may contribute to heightened discomfort and suggest that vulnerability to stress, mediated by the parasympathetic nervous system, exacerbates factor for PMS symptoms.

## 1 Introduction

Approximately 80% of women of reproductive age experience one or more physical and emotional symptoms during the premenstrual phase of the menstrual cycle (Hylan et al., 1999; Steiner et al., 2003). When these symptoms are confirmed over two or more cycles and significantly impact daily life, the condition is classified as premenstrual syndrome (PMS). PMS symptoms often worsen significantly during the late luteal phase, typically starting around 6 days before the onset of menstruation and peaking approximately 2 days before menstruation begins (Meaden et al., 2005; Pearlstein et al., 2005). These symptoms generally subside or disappear with the onset of menstruation. The most distinctive feature of PMS is the cyclical nature of its symptoms, closely associated with the menstrual cycle.

PMS symptoms are diverse, including physical symptoms such as abdominal pain, bloating, breast tenderness or swelling, and fatigue. Emotional symptoms include depressed mood or dysphoria, anxiety or tension, affective lability, and irritability. Among these, irritability is reported as the most severe complaint and tends to appear slightly earlier than other symptoms. Typically, women experience the same symptoms consistently from one cycle to the next (Bloch et al., 1997).

The diagnostic criteria for PMS are primarily based on assessing physical, psychological, and behavioral symptoms that recur in relation to the menstrual cycle. Globally, the criteria established by organizations such as the American College of Obstetricians and Gynecologists (ACOG) and Diagnostic and Statistical Manual of Mental Disorders, 5th Edition (DSM-5) are commonly used. Based on these diagnostic criteria, the premenstrual symptoms screening tool (PSST) was developed to assess symptoms severity related to PMS and premenstrual dysphoric disorder (PMDD) as well as their impact on daily life (Steiner et al., 2003). The prevalence of PMS among all women is estimated at approximately 50%, with symptoms ranging from mild to severe (Direkvand-Moghadam et al., 2014). However, the diagnosis of PMS often proves challenging as it relies on the subjective evaluation of symptoms. Additionally, many women do not seek medical consultation regarding their symptoms, leading to delayed or undiagnosed cases (del Mar Fernández et al., 2019).

PMS is influenced by menstrual cycle-related sex hormones such as estrogen and progesterone. Receptors for these hormones are abundantly expressed in brain regions such as the amygdala and hypothalamus (Del Río et al., 2018), and they are known to interact closely with neurotransmitters, significantly affecting women’s emotions and mood (Del Río et al., 2018; Fruzzetti and Fidecicchi, 2020). While these sex hormones fluctuate cyclically, they decrease sharply during the late luteal phase, leading to a significant worsening of PMS symptoms. However, the exact mechanisms underlying the development of PMS remain unclear. This is because there is no difference in blood concentrations of sex hormones, or any structural abnormalities, between women with and without PMS (Backstrom et al., 1983; Rubinow and Schmidt, 1995; Rubinow and Schmidt, 2019). Consequently, it is challenging to identify clear pathological findings specific to PMS (Gollenberg et al., 2010). Instead, women with PMS may exhibit hypersensitivity to fluctuations in sex hormone levels within the normal range (Steiner et al., 2003; Yonkers et al., 2008).

Understanding how this heightened sensitivity to hormonal fluctuations affects emotional states in women with PMS is critical. We hypothesize that women with PMS may experience difficulties in the processing of internal state information, such as sensitivity to visceral conditions and sex hormone levels, which is referred to as interoception. Interoception is involved in updating the internal model of the body and the emergence of emotions (Khalsa et al., 2018). Interoceptive accuracy is associated with the intensity of emotional experiences (Garfinkel and Critchley, 2013); interoceptive awareness significantly influences the severity of menstrual and premenstrual symptoms (Borlimi et al., 2023). It is crucial to examine women with PMS for their interoceptive accuracy and awareness to better understand the severe discomfort they experience. We assessed subjective emotional states, interoceptive accuracy, and interoceptive awareness during the late luteal phase, a period characterized by a sharp decline in sex hormone levels.

Interoceptive accuracy was assessed using the Heartbeat Counting Task (HCT; Schandry, 1981); meanwhile, interoceptive awareness, reflecting interoceptive body consciousness, was evaluated through self-reports using the Multidimensional Assessment of Interoceptive Awareness (MAIA; Mehling et al., 2012). Subjective emotional states were measured using the Positive and Negative Affect Schedule (PANAS; Watson et al., 1988) and stress scores on the Visual Analogue Scale (VAS).

Interoceptive signals are transmitted to the brain through the autonomic nervous system (ANS) and contribute to the maintenance of homeostasis (Tsakiris and Critchley, 2016). The ANS, composed of the sympathetic and parasympathetic branches, plays a crucial role in unconsciously regulating basic bodily functions (Critchley and Harrison, 2013). However, PMS has been associated with ANS imbalances, and heart rate variability (HRV) has decreased in women with severe PMS (Matsumoto et al., 2007a, 2007b). When this regulatory mechanism is disrupted, the effects can extend throughout the body, leading to the development of complex and multifaceted symptoms, such as those seen in PMS (Matsumoto et al., 2007a, 2007b). One of the exacerbating factors of PMS is stress (Gollenberg et al., 2010). Acute stress during the luteal phase in women with PMS, in addition to chronic menstrual stress, significantly exacerbates autonomic dysfunction (Meng et al., 2022). Imbalance in autonomic nervous function disrupts the proper transmission of interoceptive signals, leading to inaccurate recognition of the body’s internal states and emotional instability (Critchley and Harrison, 2013).

Thus, clarifying how stress impairs the appropriate recognition of bodily information in women with PMS is important, as this contributes to the experience of more severe negative emotions. We hypothesize that stress during the late luteal phase disrupts the transmission of interoceptive signals and impairs the stress recovery process in women with PMS through ANS imbalance. By examining changes in subjective emotions, interoceptive accuracy, and ANS activity during, before, and after stress, including the recovery period, we investigated the influences of stress in women with PMS.

## 2 Materials and methods

### 2.1 Participants

Menstrual cycles were recorded over two cycles, with healthy women aged 18–34 years participating in the study. The 90 participants comprised undergraduate students, graduate students, and non-degree students affiliated with Nagoya University, 89 of them Japanese and one non-Japanese. The non-Japanese participant and one Japanese participant withdrew, and their data were excluded from the analysis. The average age of the participants was 20.568 ± 2.577 years (PMS group 21.250 ± 3.905, without PMS group 20.268 ± 2.029); the mean age at menarche was 12.013 ± 1.354 years (PMS group 11.842 ± 2.007, without PMS group 12.085 ± 1.083). Based on PMS screening results, 21 participants were classified as with PMS, while 67 were classified as without PMS. Recruitment was conducted through the university’s participant recruitment system. The exclusion criteria comprised the use of oral contraceptives, current pregnancy or breastfeeding, currently undergoing psychiatric treatment, use of medications affecting stress reactivity, or highly irregular menstrual cycles.

Participants underwent an interview prior to the experiment to estimate their next ovulation date based on the two recorded menstrual cycles. After receiving instructions from a pharmacist on how to use the ovulation test kit (Dotest LHII Rohto Pharmaceutical), participants measured their LH surge and estimated the next menstrual onset. The laboratory experiment was conducted between 7 days and 1 day before the estimated onset of menstruation. Following the experiment, participants reported the actual onset of menstruation, which was used to confirm the luteal phase.

### 2.2 Subjective emotions

#### 2.2.1 The Positive and Negative Affect Schedule

The PANAS is designed to assess two major affective dimensions, Positive Affect and Negative Affect, and consists of 22 items designed to assess the degree of the rating of the extent to which the words describe an emotion or mood (Watson et al., 1988). The Japanese version of the PANAS, the reliability and validity of which has been confirmed, was used in the experiment (Watson and Clark, 1989). The assessment was made using a 5-point Likert scale, recognized as a standard tool for assessing emotions.

### 2.3 Interoceptive measurements

#### 2.3.1 Interoceptive accuracy

Interoceptive accuracy was assessed using the HCT, a well-established and validated method in clinical research for measuring interoceptive accuracy. Participants were instructed to focus on their heartbeat while sitting quietly in a noise-free room, and to silently count their “truly felt” heartbeats.

They were instructed not to use physical methods to detect heartbeats, such as taking a pulse or checking a watch. Heartbeats were counted during three intervals of 25 s, 35 s, and 45 s, with participants reporting the count at the end of each interval. The experimenter indicated the start and end of each counting interval using start and stop cues. Participants were unaware of the duration of the intervals or each trial’s results. The experimenter calculated the actual heart rate for each interval using electrocardiograms recorded by electrodes attached to the participants’ right and left wrists.

The HCT score was calculated as the average score across the three counting intervals, according to the following formula:


$$\begin{array}{l} \mathrm{HCT}\;\mathrm{score}=1/3\Sigma (1-\mathrm{recorded heartbeats}-\mathrm{counted heartbeats}\\ \;\;\;\;\;\;\;\;\;\;\;\;\;\;\;\;\;\;\;\;\;\;\;\;\;\;\;\;/\mathrm{recorded heartbeats}) \end{array}$$

The HCT score ranges from 0 to 1, with higher scores indicating greater accuracy in heartbeat perception (Schandry, 1981).

#### 2.3.2 Interceptive awareness

Interceptive awareness was assessed using the MAIA questionnaire, which assesses interoceptive awareness (awareness of internal bodily sensations and states) in a multidimensional manner. It consists of eight subscales, each evaluating different aspects of interoceptive awareness: Noticing: Awareness of uncomfortable, comfortable, and neutral body sensations; Not-distracting: Tendency not to ignore or distract oneself from sensations of pain or discomfort; Not-worrying: Tendency not to worry or experience emotional distress with sensations of pain or discomfort; Attention regulation: Ability to sustain and control attention to body sensations; Emotional awareness: Awareness of the connection between body sensations and emotional states; Self-regulation: Ability to regulate distress by paying attention to body sensations; Body listening: Active listening to the body for insight; and Trusting: Experience of one’s body as safe and trustworthy. Each item is rated on a 6-point Likert scale ranging from “0 = never” to “5 = always.” Higher scores indicate higher levels of interoceptive awareness (Mehling et al., 2012). In this study, the total scores of the eight subscales of the MAIA were used as the MAIA total scores. The Japanese version of the MAIA was used in the experiment, and its reliability and validity have been confirmed (Shoji et al., 2018).

### 2.4 Autonomic nervous system activity

ANS was estimated through HRV, which represents the fluctuations in heartbeat intervals (RR intervals). Heart rate was measured using the BIOPAC system’s electrocardiogram (ECG100C) at 1,000 Hz sampling rate, and data were collected with the MP150 series. Electrodes (F-Bitrode) were attached to the participants’ right and left wrists, and measurements were taken in a seated resting position. Measurements were conducted in a shielded room adjacent to the experimental room, in a quiet and temperature-controlled environment, where participants were in a state of minimal physical and mental stress. The collected data were processed using AcqKnowledge software, which detected RR intervals. The data were then corrected using a 5 Hz high-pass filter through the transform function.

#### 2.4.1 Data preprocessing of RR intervals

The procedures applied to RR intervals containing ectopic beats or artifacts are as follows: (1) replacing the interval with the mean of the surrounding RR intervals, (2) adjusting it to be below a value equal to the mean RR multiplied by a constant *M*, and (3) replacing it if it differed by more than 30% from the previous RR interval. The mean RR was calculated as a moving average centered on the beats requiring correction, with a range of ±3 beats considered normal. The analysis was performed using a sequence of 300 beats with a 5-beat overlap (Magagnin et al., 2011). Data processing and analysis were performed using Python 3.11.4 on Jupyter Notebook 7.2.2.

#### 2.4.2 Heart rate variability

Heart rate (HR) and RR intervals were utilized. HRV was analyzed based on the RR intervals. In the frequency-domain HRV indices, high frequency (HF; 0.15–0.4 Hz) primarily reflects vagal nerve activity, serving as an indicator of parasympathetic modulation. Low frequency (LF; 0.04–0.15 Hz) is mainly associated with sympathetic nervous activity but includes some parasympathetic influences; it is affected by blood pressure regulation. The LF/HF ratio evaluates the balance between sympathetic and parasympathetic activity in the ANS. It is considered an indicator of neural modulation, reflecting changes relative to the tonic level of neural activity about its mean value (La Rovere et al., 2020). A higher LF/HF ratio indicates sympathetic dominance, whereas a lower ratio suggests parasympathetic dominance. Very low frequency (VLF) represents frequencies below 0.04 Hz. Total power (TP) reflects overall autonomic regulatory function.

In the time-domain HRV indices, including the standard deviation of NN intervals (SDNN), root mean square of successive differences (RMSSD), and pNN50, were utilized. SDNN represents the standard deviation of normal-to-normal RR intervals (NN intervals) and is used to evaluate the overall autonomic regulation capacity. RMSSD is a time-domain HRV metric that reflects the root mean square of successive differences between adjacent RR intervals. pNN50 indicates the percentage of successive RR intervals that differ by more than 50 ms. High RMSSD and pNN50 values reflect a parasympathetic-dominant state. The analysis was performed using Kubios software (Kubios HRV Standard 3.5.0) to calculate frequency-domain and time-domain parameters.

#### 2.4.3 Stationary test

The stationarity of HRV refers to the condition in which the statistical properties of HRV data remain constant over time. It was assessed using the Augmented Dickey–Fuller (ADF) test. The ADF test is a statistical method used to evaluate the presence of a unit root in time-series data and determine whether the data exhibit stationarity. In this study, the ADF test was performed after applying a moving average filter, and stationarity was confirmed when the *p*-value was less than 0.05 (Sides et al., 2023).

### 2.5 Procedures

#### 2.5.1 PMS screening

PMS screening was conducted using the premenstrual symptoms screening tool (PSST) (Steiner et al., 2003). The Japanese version of the PSST was adapted, whose validity and reliability have been confirmed by Miyaoka (2009). The PSST assesses the impact of premenstrual symptoms on daily life, and can diagnose PMDD, a severe form of PMS. It consists of items that evaluate premenstrual symptoms and their impact on daily life. The items related to premenstrual symptoms assess both psychological and physical symptoms and are rated on a 4-point Likert scale: “not at all,” “mild,” “moderate,” and “severe.” The items assessing the impact on daily life evaluate how these symptoms affect daily activities, work, and interpersonal relationships using the same 4-point Likert scale. The score obtained from the items related to premenstrual symptoms on the PSST was referred to as the PMS score in this study. Higher PMS scores indicate more severe premenstrual symptoms. The distribution of PMS score is available in Supplementary Figure S1.

#### 2.5.2 Experimental procedures

##### 2.5.2.1 Preparation

The experimental room was maintained in a quiet and well-ventilated state. Upon entering the room, participants were instructed to gargle, and their body temperature was measured. Before beginning the experiment, informed consent was obtained from all participants. A portable heart rate monitor was affixed to the left subclavicular region of each participant.

##### 2.5.2.2 During the experiment

The experiment was structured into three phases, waiting time phase and stress induction phase: Phase 1 (P1) occurred before inducing stress, Phase 2 (P2) spanned from 0 to 30 min post-stress induction, waiting time (WT) phase covered 30 to 60 min post-stress induction, and Phase 3 (P3) encompassed 60 to 90 min post-stress induction.

In P1, P2, and P3, participants underwent blood pressure measurements and an HCT, provided saliva samples, and completed questionnaires. Additionally, their heart rates were continuously monitored for 7 min while they engaged in a cognitive task. In WT, participants completed a questionnaire and provided a saliva sample. Upon completion of the experiment, the portable heart rate monitor was removed, and a debriefing session was conducted. The procedure followed the principles of the Declaration of Helsinki and was approved by the Ethics Committee of the Department of Psychology at Nagoya University.

**Figure 1:**
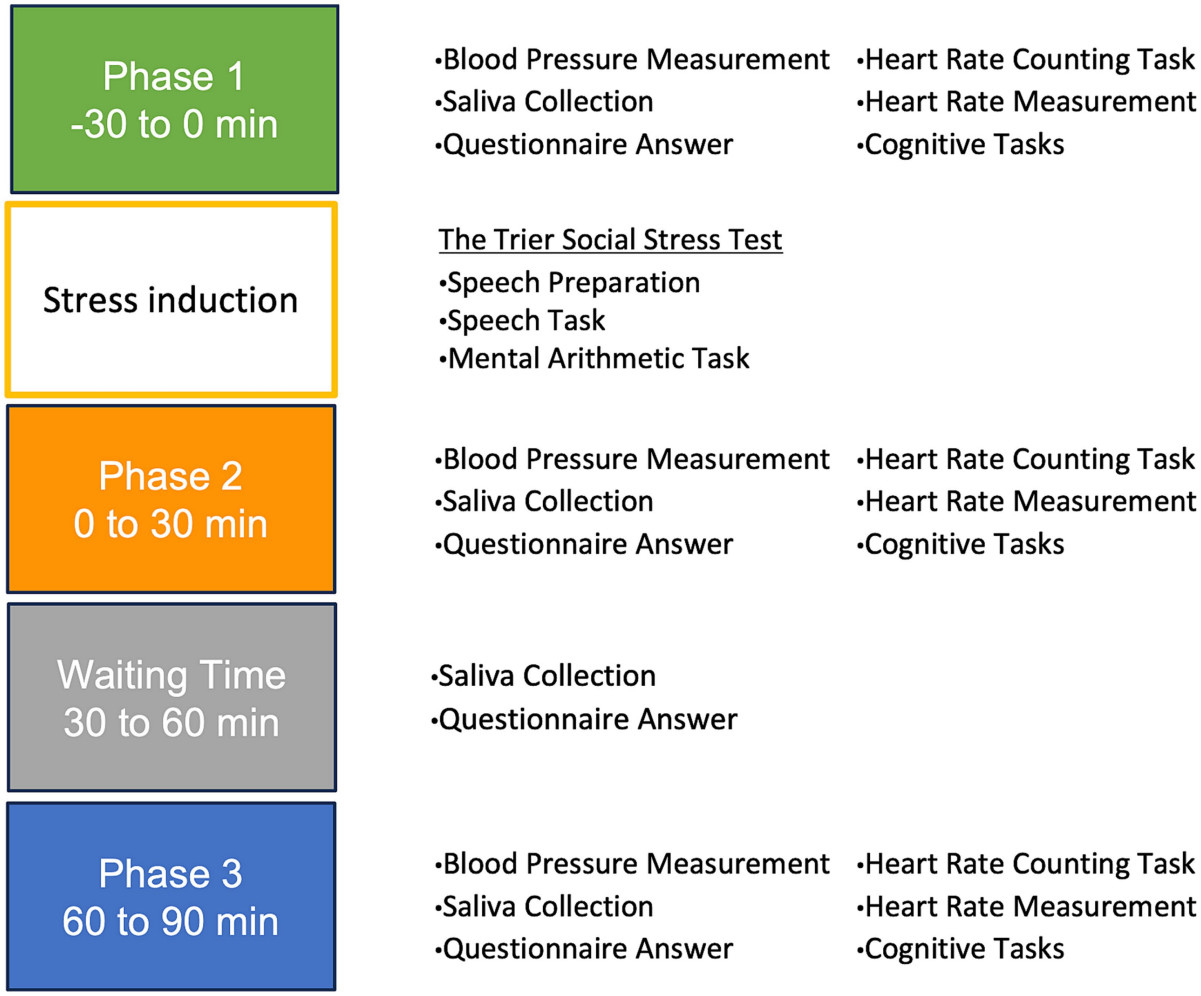
The flow of the laboratory experiment. Phase 1: −30 to 0 min (baseline/pre-stress). Phase 2: 0 to 30 min (immediately post-stress). Phase 3: 30 to 60 min (waiting period). Phase 4: 60 to 90 min (stress recovery).

This study conducted a series of experiments to evaluate interoceptive accuracy and its impact on ANS activity during stress. The flow of the laboratory experiments is shown in Figure 1.

#### 2.5.3 Stress induction

Stress was induced by the Trier Social Stress Test (the TSST) in the stress induction phase, with all instructions provided in Japanese (Kirschbaum et al., 1993; Shimizu, 2016). The TSST protocol consisted of two main phases: preparation and task execution, with a speech task and a mental arithmetic task to be performed.

##### 2.5.3.1 Preparation phase

Participants were taken to a room and briefed on the task. They were asked to give a good description of themselves and given 5 min to prepare.

##### 2.5.3.2 Task execution phase

Speech task (5 min): Participants faced a white-coated judge who maintained a neutral or somewhat critical expression. Participants were asked to give a prepared speech. They were informed that their speeches would be recorded, and their content would be evaluated by the judges. If they finished early, they were encouraged to continue speaking on the same topic. Mental arithmetic task (5 min): Immediately following the speech task, participants were asked to perform a continuous subtraction task. They were given a starting number (e.g., 2,097) and asked to keep subtracting 13 from it. They were asked to do this as quickly and accurately as possible and to start over from the original number if they made a mistake. Both were designed to induce stress.

#### 2.5.4 Subjective stress

The subjective stress was measured using the Visual Analog Scale (VAS), with all instructions provided in Japanese. Participants in the experiment marked a point corresponding to their current level of stress on a horizontal straight line measuring 8 cm. The left end of the scale represented “0 = not stressed at all,” and the right end represented “8 = very stressed.”

### 2.6 Data statistics

The mean values of subjective emotions, interoceptive indices, and ANS indices in P1 were evaluated between the PMS and without PMS groups using *t*-tests. To evaluate the mean values of subjective emotions, interoceptive accuracy, and HRV indices across P1, P2, and P3, a mixed-design ANOVA was conducted. In this analysis, group (PMS and without PMS) was set as a between-subjects factor, and Time (P1, P2, and P3) was set as a within-subjects factor. For indices where the ANOVA results indicated differences in mean values across time, Holm’s method for multiple comparisons was applied to determine whether each index showed significant differences between times (P1 vs. P2, P1 vs. P3, and P2 vs. P3). For subjective emotional states, additional comparisons were made between P1 vs. WT, P2 vs. WT, and WT vs. P3. Multiple comparison results included the following parameters: mean difference (MD), standard error (SE), 95% lower confidence limit (95% lower CI), 95% upper confidence limit (95% upper CI), and adjusted *p*-value (Adj. *p*; Holm).

The degree of parasympathetic activity response was indicated using *D*-values (Meng et al., 2022). The *D*-value immediately after stress was calculated as the difference [Δ(P2 − P1)] between data in the baseline (P1) and data immediately after stress (P2). The *D*-value during the recovery period was calculated as the difference [Δ(P3 − P1)] between data in the baseline phase (P1) and data during the recovery phase (P3). The group factor (PMS and without PMS) was treated as a between-subject factor, while time [Δ(P2 − P1) and Δ(P3 − P1)] was treated as a within-subject factor. A *p*-value of less than 0.05 was considered statistically significant, with ^*^p < 0.05, ^**^p < 0.01, and ^***^p < 0.001. Cohen’s *d* was used to calculate effect sizes for *t*-tests, *R*^2^ for regression analyses, and *η*^2^ for ANOVA. “n.s.” denotes non-significant results. Statistical analyses and data processing were performed using HAD18 (Shimizu, 2016).

## 3 Results

### 3.1 Pre-stress

**Figure 2:**
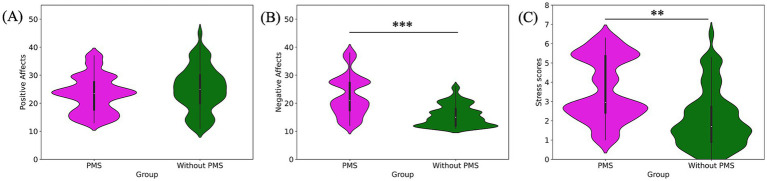
Pre-stress comparison of subjective emotional states: positive affect **(A)**, negative affect **(B)**, and stress scores **(C)**. Negative affect and stress scores were significantly higher in the PMS group.

**Figure 3:**
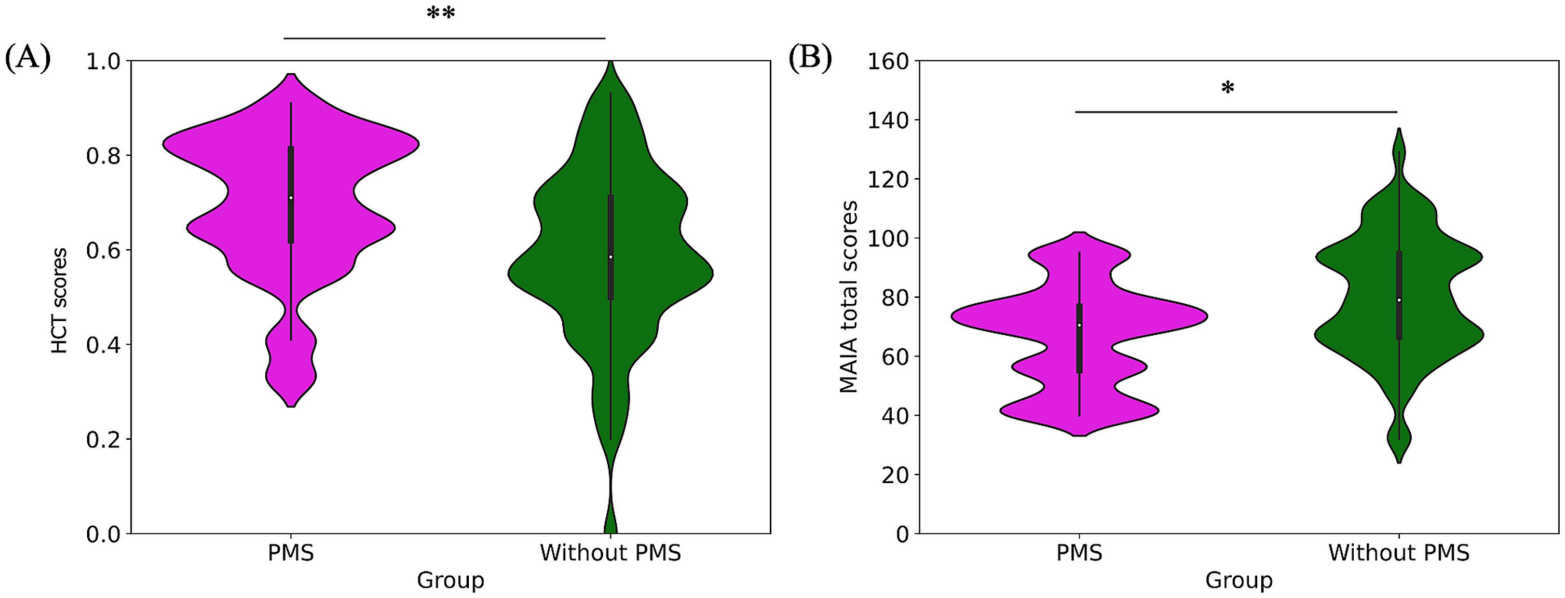
Pre-stress comparison of interoceptive measures: HCT scores **(A)** and MAIA total scores **(B)**. The PMS group had significantly higher HCT scores but lower MAIA total scores.

**Figure 4:**
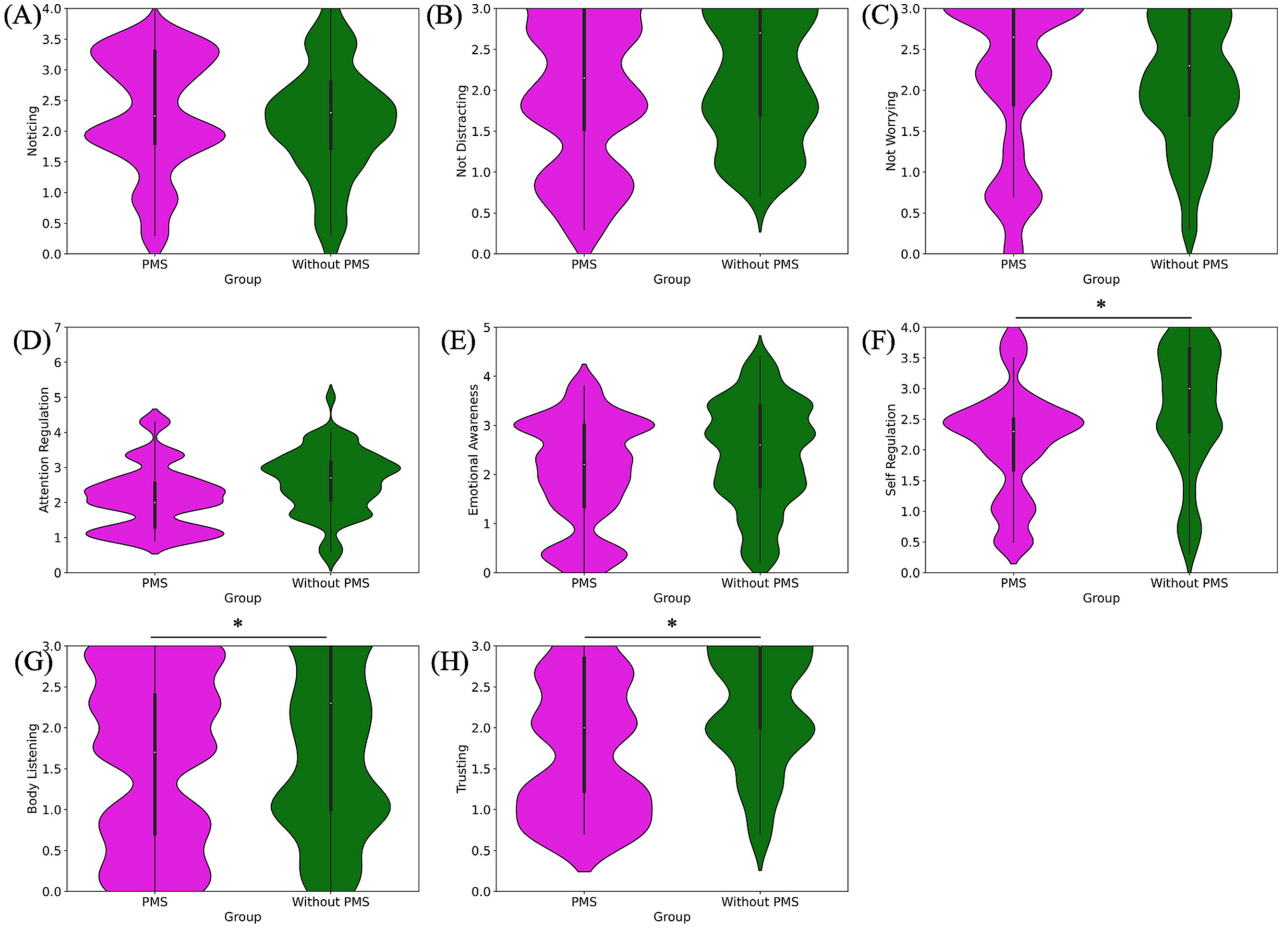
Pre-stress comparison of MAIA subscale scores: noticing **(A)**, not-distracting **(B)**, not-worrying **(C)**, attention regulation **(D)**, emotional awareness **(E)**, self-regulation **(F)**, body listening **(G)** and trusting **(H)**. Self-regulation, body listening, and trusting scores were significantly lower in the PMS group.

**Figure 5:**
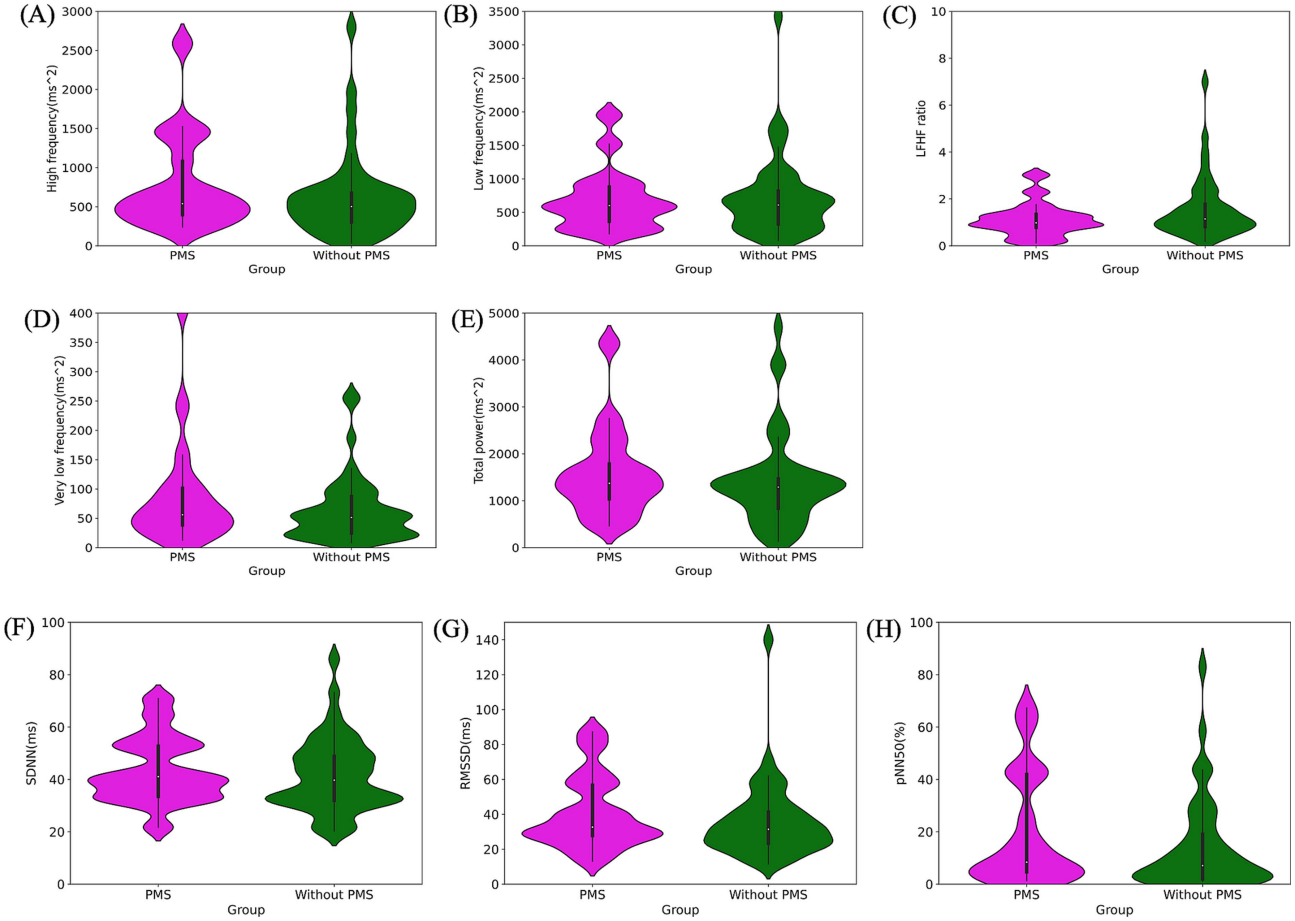
Pre-stress comparison of autonomic nervous system activity: frequency domain: high frequency (ms^2^) **(A)**, low frequency (ms^2^) **(B)**, LF/HF ratio **(C)**, very low frequency (ms^2^) **(D)**, total power (ms^2^) **(E)**. Time domain: SDNN (ms) **(F)**, RMSSD (ms) **(G)**, pNN50 (%) **(H)**. All indices showed no significant differences between the PMS group and the without PMS group.

**Figure 6:**
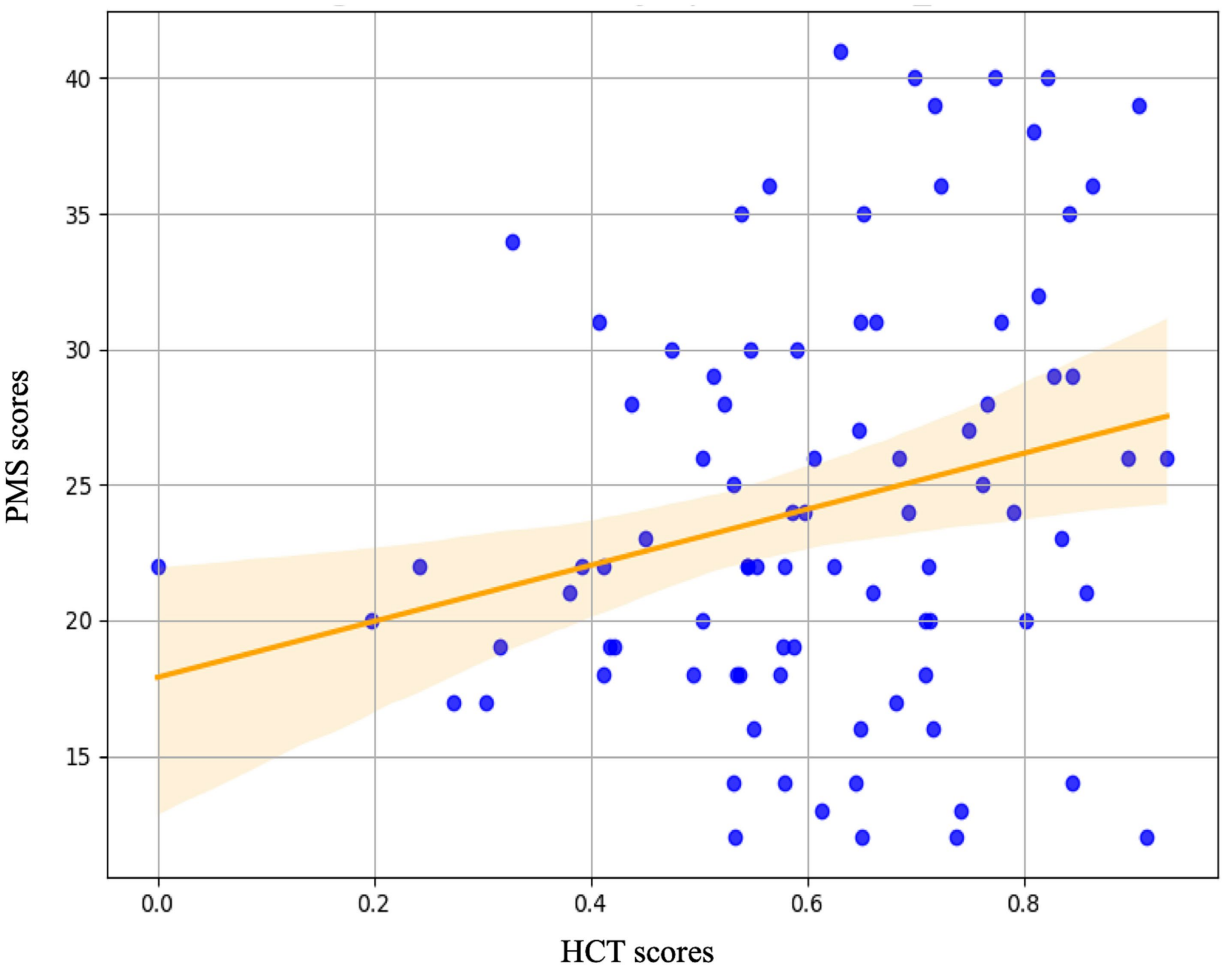
The regression analysis with HCT scores indices during the pre-stress baseline (Phase 1) as predictor variables and PMS scores as the outcome variable. The analysis revealed that the Heartbeat Counting Task score was a significant predictor of PMS scores.

The results from P1, representing the pre-stress condition, are presented in Figures 2–6 and summarized in Tables 1, 2. An independent *t*-test was performed to compare the PMS group with the without PMS group.

**Table 1:** Results of the independent *t*-test between the PMS group and the without PMS group during the pre-stress baseline (Phase 1).

	Indices	*t* (df)	*p*-value	Effect size (*d*)	Significant
Subjective emotions	Positive affects	*t* (36.146) = 1.012	0.318	0.233	n.s.
	Negative affects	*t* (22.048) = 4.099	0.000	1.442	***
	Stress scores	*t* (28.525) = 3.705	0.001	0.994	***
Interoceptive measurements	HCT scores	*t* (35.287) = 2.539	0.016	0.592	*
	MAIA total scores	*t* (30.460) = 2.892	0.007	0.698	**
MAIA subscale	Noticing	*t* (28.047) = 0.195	0.846	0.051	n.s.
	Not-distracting	*t* (28.568) = 1.521	0.139	0.394	n.s.
	Not-worrying	*t* (24.831) = 0.119	0.907	0.035	n.s.
	Attention regulation	*t* (26.267) = 1.779	0.087	0.473	n.s.
	Emotional awareness	*t* (27.800) = 1.526	0.138	0.404	n.s.
	Self-regulation	*t* (38.691) = 2.585	0.014	0.563	*
	Body listening	*t* (36.562) = 2.337	0.025	0.521	*
	Trusting	*t* (28.468) = 2.698	0.012	0.701	*
Autonomic measurements	HR	*t* (28.941) = 1.089	0.285	0.306	n.s.
	RR interval	*t* (26.596) = 1.088	0.286	0.320	n.s.
Frequency domain	HF	*t* (25.019) = 1.096	0.283	0.334	n.s.
	LF	*t* (36.305) = 0.187	0.853	0.047	n.s.
	LF/HF	*t* (49.998) = 1.610	0.114	0.357	n.s.
	VLF	*t* (19.870) = 1.118	0.277	0.402	n.s.
	TP	*t* (29.077) = 0.692	0.494	0.194	n.s.
Time domain	SDNN	*t* (31.037) = 0.501	0.620	0.136	n.s.
	RSMDD	*t* (29.127) = 0.880	0.386	0.246	n.s.
	pNN50	*t* (24.121) = 1.044	0.307	0.325	n.s.

**Table 2:** The regression analysis with interoceptive-related indices before stress (Phase 1) as predictor variables and PMS scores as the outcome variable.

Predictor variable	*t* (df)	*p*-value	*R* ^2^	*b*	SE	Significant
HCT scores	*t* (86) = 2.275	0.025	0.057	0.005	0.002	*
MAIA total scores	*t* (83) = −1.046	0.299	0.013	−0.042	0.040	n.s.
MAIA subscale						
Noticing	*t* (85) = 0.740	0.461	0.006	0.570	0.770	n.s.
Not-distracting	*t* (85) = −1.568	0.121	0.028	−1.160	0.740	n.s.
Not-worrying	*t* (85) =0.375	0.708	0.002	0.329	0.877	n.s.
Attention regulation	*t* (84) = −0.079	0.937	0.000	−0.071	0.897	n.s.
Emotional awareness	*t* (85) = −0.905	0.368	0.010	−0.680	0.751	n.s.
Self-regulation	*t* (84) =0.939	0.350	0.010	−0.715	0.761	n.s.
Body listening	*t* (85) = −0.820	0.414	0.008	−0.523	0.637	n.s.
Trusting	*t* (85) = −1.674	0.098	0.032	−1.172	0.700	n.s.

The findings for subjective emotional states revealed that the PMS group had significantly higher scores for subjective negative affects (^***^p = 0.000) and subjective stress (^**^p = 0.001). By contrast, no significant difference was observed in subjective positive affects (*p* = 0.318). The comparison of subjective emotional states was shown in Figure 2 and Table 1.

Regarding interoceptive measurements, the PMS group demonstrated significantly higher scores on the HCT compared to the without PMS group (^**^p = 0. 005). Conversely, the MAIA total scores were significantly lower in the PMS group than in the without PMS group (^*^p = 0.016). The comparison of interoceptive measures was shown in Figure 3 and Table 1.

The results of the eight subscales of the MAIA are as follows. The PMS group exhibited significantly lower scores for self-regulation (^*^p = 0.012), body listening (^*^p = 0.018), and trusting (^*^p = 0.017) compared to the without PMS group. However, no significant differences were observed for noticing (*p* = 0.786), not-distracting (*p* = 0.154), not-worrying (*p* = 0.607), attention regulation (*p* = 0.077), and emotional awareness (*p* = 0.109). The comparison of MAIA subscale scores are shown in Figure 4 and Table 1.

The results of the ANS activity analysis are as follows. No significant differences were observed in HR (*p* = 0.285) and RR interval (*p* = 0.286). The frequency domain analysis revealed no significant differences between the two groups for HF (*p* = 0.283), LF (*p* = 0.853), HF/LF (*p* = 0.114), VLF (*p* = 0.277), and TP (*p* = 0.494). Similarly, the time-domain analysis exhibited no significant differences in SDNN (*p* = 0.620), RMSSD (*p* = 0.386), and PNN50 (*p* = 0.307). The comparison of ANS activity, particularly in the frequency and time domains, is presented in Figure 5 and Table 1.

The results of the simple regression analysis are as follows. Regression analysis was performed utilizing interoceptive measures as predictor variables and PMS scores as the dependent variable. Interoceptive measures included the HCT score, the MAIA total score, and its eight subscales. The HCT score significantly predicted PMS scores (^*^p = 0.025). Conversely, neither the MAIA total score (*p* = 0.299), nor its eight subscales, including noticing (*p* = 0.461), not-distracting (*p* = 0.121), not-worrying (*p* = 0.708), attention regulation (*p* = 0.937), emotional awareness (*p* = 0.368), self-regulation (*p* = 0.350), body listening (*p* = 0.414), and trusting (*p* = 0.098), significantly predicted PMS scores. The results of the simple regression analysis are detailed in Figure 6 and Table 2.

### 3.2 Effects of stress: pre-stress, post-stress, and recovery

**Figure 7:**
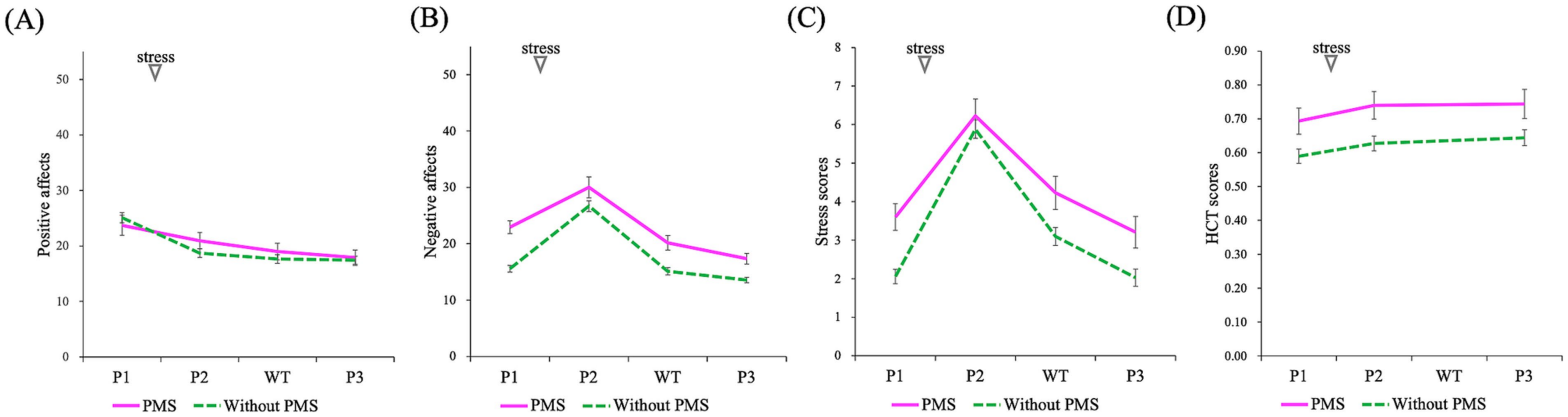
Comparison of the effects of stress induction on subjective emotions and HCT scores (P1: baseline/pre-stress, P2: immediately post-stress, P3: stress recovery): positive affects **(A)**, negative affects **(B)**, stress scores **(C)**, HCT scores **(D)**. A mixed-design ANOVA revealed that the PMS group exhibited significantly higher negative affect and stress scores. Additionally, a significant main effect of time (P1, P2, WT, and P3) was observed across all indices whereas no significant interactions were found. However, WT represents waiting time and was measured using subjective emotions.

**Figure 8:**
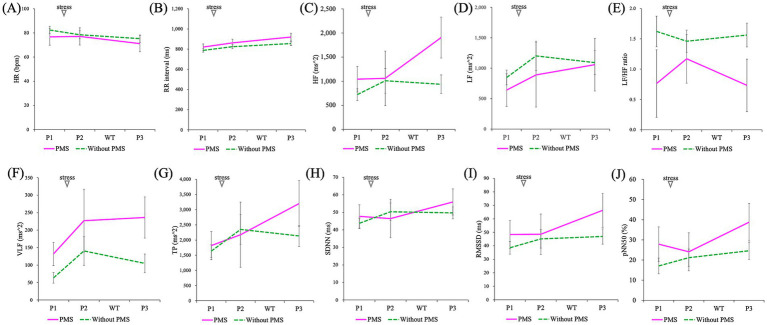
Comparison of the effects of stress induction on autonomic nervous system activity (P1: baseline/pre-stress, P2: immediately post-stress, and P3: stress recovery): HR **(A)**, RR interval **(B)**, HF **(C)**, LF **(D)**, LF/HF ratio **(E)**, VLF **(F)**, TP **(G)**, SDNN **(H)**, RMSSD **(I)**, and pNN50 **(J)**. A mixed-design ANOVA showed that all indices showed no significant differences between the groups and no significant interaction effects. However, a significant main effect of time (P1, P2, and P3) was observed for HR, RR interval, HF, RMSSD, and pNN50.

**Figure 9:**
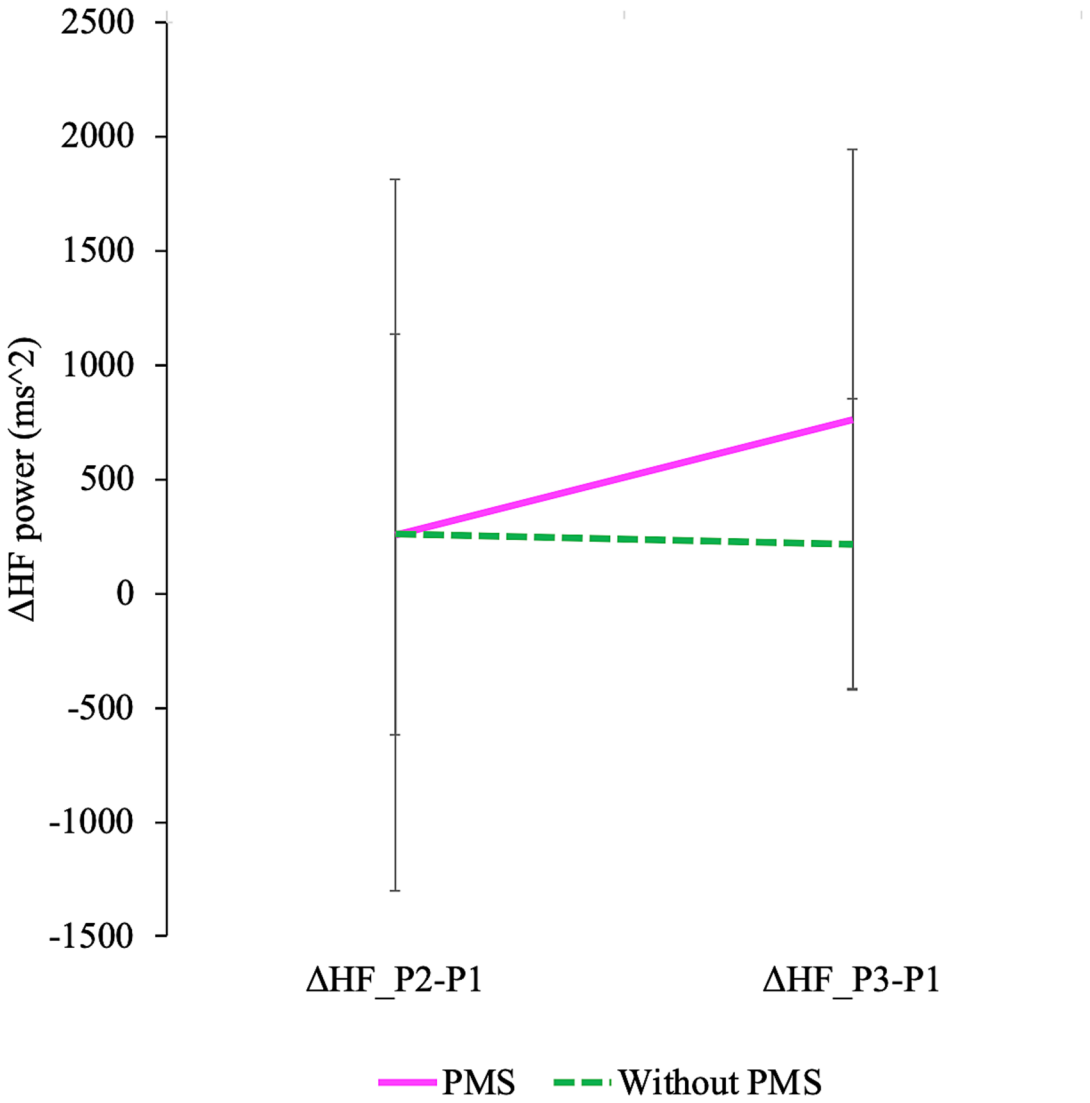
The degree of changes in HF is shown. Delta HF_P2 − P1 represents the difference in HF between P2 and P1, while delta HF_P3 − P1 represents the difference in HF between P3 and P1 (P1: baseline/pre-stress, P2: immediately post-stress, and P3: stress recovery). A mixed design ANOVA revealed a significant main effect of time (delta HF_P2 − P1 and delta HF_P3 − P1) was observed. Additionally, a significant interaction effect was observed.

The results from the pre-stress, post-stress, and recovery phases, representing the effects of stress, are presented in Figures 7–9 and summarized in Table 3. A mixed analysis of variance (mixed ANOVA) was conducted for subjective emotional states. Significant group differences were observed for subjective negative affects (^***^p = 0.000) and subjective stress scores (^**^p = 0.004), but no significant difference was observed in subjective positive affects (*p* = 0.652). Significant time effects were detected for subjective negative affects (^***^p = 0.000), subjective stress scores (^***^p = 0.000), and subjective positive affects (^***^p = 0.000). However, no significant interactions were observed for subjective negative affects (*p* = 0.099), subjective stress scores (*p* = 0.105), or subjective positive affects (*p* = 0.117).

**Table 3:** Results of mixed ANOVA for the PMS group and the Without PMS group across the pre-stress, post-stress, and recovery phases.

	Indices	*F* (df)	*p*-value	Effect size (*η*^2^)	Significant
(A) Subjective emotions and interoceptive measurement					
Group (main effect)	Positive affects	*F* (1, 84) = 0.205	0.652	0.002	n.s.
	Negative affects	*F* (1, 84) = 18.725	0.000	0.182	***
	Stress scores	*F* (1, 86) = 8.797	0.004	0.093	**
	HCT scores	*F* (1, 86) = 5.737	0.019	0.063	*
Time (main effect)	Positive affects	*F* (3, 252) = 32.489	0.000	0.279	***
	Negative affects	*F* (3, 252) = 91.988	0.000	0.523	***
	Stress scores	*F* (3, 258) = 82.943	0.000	0.491	***
	HCT scores	*F* (2, 172) = 9.028	0.000	0.095	***
Group × time (interaction)	Positive affects	*F* (3, 252) = 2.085	0.117	0.024	n.s.
	Negative affects	*F* (3, 252) = 2.360	0.099	0.027	n.s.
	Stress scores	*F* (3, 258) = 2.118	0.105	0.024	n.s.
	HCT scores	*F* (2, 172) = 0.129	0.850	0.002	n.s.
(B) Autonomic measurements					
Group (main effect)	HR	*F* (1, 33) = 0.250	0.621	0.008	n.s.
	RR interval	*F* (1, 33) = 0.502	0.484	0.008	n.s.
	HF	*F* (1, 33) = 1.134	0.295	0.033	n.s.
	LF	*F* (1, 33) = 0.197	0.660	0.006	n.s.
	LF/HF	*F* (1, 33) = 2.472	0.125	0.070	n.s.
	VLF	*F* (1, 33) = 3.948	0.055	0.107	n.s.
	TP	*F* (1, 33) = 0.220	0.642	0.007	n.s.
	SDNN	*F* (1, 33) = 0.059	0.809	0.002	n.s.
	RSMDD	*F* (1, 33) = 0.688	0.413	0.020	n.s.
	pNN50	*F* (1, 33) = 1.018	0.320	0.030	n.s.
	ΔHF: Δ(P2 − P1) − Δ(P3 − P1)	*F* (1, 33) = 0.223	0.640	0.007	n.s.
Time (main effect)	HR	*F* (2, 66) = 11.465	0.000	0.258	***
	RR interval	*F* (2, 66) = 12.771	0.000	0.279	***
	HF	*F* (2, 66) = 3.731	0.037	0.102	*
	LF	*F* (2, 66) = 1.938	0.159	0.055	n.s.
	LF/HF	*F* (2, 66) = 0.228	0.797	0.007	n.s.
	VLF	*F* (2, 66) = 2.049	0.140	0.058	n.s.
	TP	*F* (2, 66) = 2.957	0.069	0.082	n.s.
	SDNN	*F* (2, 66) = 2.216	0.123	0.063	n.s.
	RSMDD	*F* (2, 66) = 5.032	0.012	0.132	*
	pNN50	*F* (2, 66) = 4.970	0.014	0.131	*
	ΔHF: Δ(P2 − P1) − Δ(P3 − P1)	*F* (1, 33) = 5.118	0.030	0.134	*
Group × time (interaction)	HR	*F* (2, 66) = 1.289	0.281	0.038	n.s.
	RR interval	*F* (2, 66) = 1.135	0.322	0.033	n.s.
	HF	*F* (2, 66) = 2.698	0.084	0.076	n.s.
	LF	*F* (2, 66) = 0.287	0.720	0.009	n.s.
	LF/HF	*F* (2, 66) = 0.774	0.466	0.023	n.s.
	VLF	*F* (2, 66) = 0.248	0.767	0.007	n.s.
	TP	*F* (2, 66) = 1.354	0.264	0.039	n.s.
	SDNN	*F* (2, 66) = 1.218	0.300	0.036	n.s.
	RSMDD	*F* (2, 66) = 1.734	0.188	0.050	n.s.
	pNN50	*F* (2, 66) = 1.491	0.235	0.043	n.s.
	ΔHF: Δ(P2 − P1) − Δ(P3 − P1)	*F* (1, 33) = 7.201	0.011	0.179	*

For subjective emotions (A), heart rate counting tasks (A), and autonomic nervous system indices (B), the results of the mixed ANOVA with group (PMS vs. without PMS) and time (Phase 1: baseline/pre-stress, Phase 2: immediately post-stress, waiting time for subjective emotions, Phase 3: stress recovery). The analysis of delta HF (B) in the mixed ANOVA, which examined the degree of HF changes between Phase 2 and Phase 1, as well as between Phase 3 and Phase 1, showed significant time effects and interaction effects. The results suggest that the PMS group exhibited increased HF during the stress recovery phase.

For interoceptive measurements, the HCT exhibited significant primary effects for group (^*^p = 0.019) and time (^***^p = 0.000), but no significant interaction effect (*p* = 0.850).

The results of the ANOVA evaluating the effects of stress are shown in Figure 7 and Table 3.

Regarding ANS indicators, HR revealed no significant group differences (*p* = 0.621) but exhibited significant changes over time (^***^p = 0.000), with no significant interaction effects (*p* = 0.281). Similarly, for RR intervals, no main effect of group (*p* = 0.484) was found, but a main effect of time (^***^p = 0.000) was observed without interaction effects (*p* = 0.322).

In the frequency domain analysis, no significant group differences were observed for HF (*p* = 0.295), LF (*p* = 0.660), LF/HF (*p* = 0.125), VLF (*p* = 0.055), or TP (*p* = 0.642). However, a significant time effect was detected for HF (*p* = 0.037), while no significant differences were noted for LF (*p* = 0.159), LF/HF (*p* = 0.797), VLF (*p* = 0.140), or TP (*p* = 0.069). Interaction effects for any of the frequency domain indicators were also significant, including HF (*p* = 0.084), LF (*p* = 0.720), LF/HF (*p* = 0.466), VLF (*p* = 0.767), and TP (*p* = 0.264).

In the time-domain analysis, no significant differences were observed between the groups for SDNN (*p* = 0.809), RMSSD (*p* = 0.413), or pNN50 (*p* = 0.320). Significant time effects were observed for RMSSD (*p* = 0.012) and pNN50 (*p* = 0.014), but not for SDNN (*p* = 0.123). Interaction effects in the time domain were not significant for SDNN (*p* = 0.300), RMSSD (*p* = 0.188), or pNN50 (*p* = 0.235).

The effects of stress on the ANS were analyzed using ANOVA and are presented in Figure 8 and Table 3; Supplementary Table S1.

The degree of HF responses was assessed using Δ values (Meng et al., 2022). A main effect of time and an interaction between group and time were identified while no significant differences between groups were observed. ΔHF(P3 − P1) was greater than ΔHF(P2 − P1), and this effect was observed in the PMS group. The degree of changes in HF is shown in Figure 9 and Table 3.

The stationarity test results showed that stationarity was significant in the PMS group for P1 (85%), P2 (50%), and P3 (60%), and in the without PMS group for P1 (65%), P2 (71%), and P3 (62%).

## 4 Discussion

In this study, we compared subjective emotional states, interoceptive sensations, and ANS indicators between the PMS group and the without PMS group during the luteal phase. Additionally, we examined the effects of the TSST on stress responses and their recovery. The results were discussed to explore the characteristics of PMS in the luteal phase and the relationship between PMS and stress.

Based on the screening results of the study participants, 23.6% were identified as having PMS. The prevalence of PMS among all women is estimated to be around 50%, although this rate varies depending on biological factors such as social environment and age (Direkvand-Moghadam et al., 2014). In this study, the PSST was used for PMS screening. The PSST consists of two components: the severity of physical and emotional symptoms (measure of severity), and the impact of premenstrual symptoms on daily life (impact of premenstrual symptoms) (Steiner et al., 2003). The score derived from the severity of physical and emotional symptoms is referred to as the “PMS score,” with higher scores indicating more severe premenstrual symptoms. The distribution of PMS scores among the study participants ranged from a minimum of 12 to a maximum of 42 (Supplementary Figure S1).

Phase 1 corresponds to the baseline before stress. In this study, the measurements of psychological and physiological indices in women with PMS and those without PMS were compared. The results of Phase 1 showed no significant differences in positive affect states between the two groups. However, negative affect states and subjective stress levels were significantly higher in women with PMS. These findings are largely consistent with previous research (Liu et al., 2017), supporting the accurate assessment of the unpleasant symptoms experienced by women with PMS during the luteal phase and the reliable identification of PMS.

Measurements of interoception were conducted using the HCT to assess interoceptive accuracy and the MAIA to evaluate interoceptive awareness. The results showed that these two scores exhibited opposite trends. Specifically, the PMS group scored higher on the HCT but lower on the MAIA compared to the without PMS group. These findings suggest that women with PMS demonstrate higher interoceptive accuracy but lower interoceptive awareness.

Interoceptive accuracy is associated with the intensity of emotional experiences (Wiens et al., 2000). This relationship arises because improved detection of bodily information enables a clearer understanding of one’s emotions (Nord and Garfinkel, 2022). Moreover, higher interoceptive accuracy facilitates better emotion regulation in response to negative affects (Füstös et al., 2013; Pollatos et al., 2014; Kever et al., 2015).

By contrast, low interoceptive awareness has been reported to reduce bodily awareness and trust, making it harder to recognize one’s emotions. This, in turn, may hinder effective emotional processing and increase the likelihood of chronic stress (Leech et al., 2024). In this study, the PMS group showed opposite trends in the results of the HCT and MAIA. This aligns with the finding that beliefs about interoceptive aptitude do not necessarily match actual interoceptive accuracy. Furthermore, this mismatch suggests a maladaptive state in terms of mental health (Garfinkel et al., 2015; Garfinkel et al., 2016; Nord and Garfinkel, 2022).

No significant differences were found in ANS activity, responsible for transmitting interoceptive signals, between the two groups. The lack of attenuation in power spectral components during the luteal phase among PMS group in this study may be attributed to differences in experimental methods, particularly in the definition and determination of menstrual cycle phases and participant selection criteria (Matsumoto et al., 2007a, 2007b). In Matsumoto’s study, PMS was identified using a questionnaire that assessed physical and psychological complaints associated with the menstrual cycle, and comparisons were made with PMDD, a more severe form of PMS. Differences in screening methods, including the use of the PSST in this study and the more detailed classification of premenstrual symptoms by severity, are likely to have significantly influenced the results.

Finally, the absence of significant differences in ANS activity between the two groups highlights that women with PMS experience a mismatch between two distinct dimensions: interoceptive accuracy and interoceptive awareness. The mismatch between interoceptive predictions and actual interoceptive signals generates prediction errors and contributes to the emergence of emotions (Friston, 2010). Emotions and affective sensory states are believed to arise from interoceptive states of physiological arousal, as well as the precision and predictability of these signals (Barrett and Simmons, 2015; Inui, 2018).

This suggests that women with PMS may experience severe discomfort during the late luteal phase, such as depressed mood or dysphoria, anxiety or tension, affective lability, and irritability. This may be due to their ability to accurately perceive hormonal fluctuations while having reduced awareness of interoceptive sensations.

We conducted a simple regression analysis with PMS scores, which indicate the severity of PMS symptoms, as the dependent variable, and scores measuring interoceptive awareness as the independent variables. The independent variables included the HCT score, the total score of the MAIA, and its eight subscales. As a result, the predictor of PMS symptoms was identified as the HCT score. These results suggest that in predicting PMS symptoms, the objective perception of physiological body signals (as measured by the HCT) is important, while subjective interoceptive awareness (as assessed by MAIA) does not have a direct impact. Therefore, it is considered crucial to focus specifically on the “accuracy” aspect of interoceptive awareness to understand symptoms and mechanisms related to PMS.

The results of the simple regression analysis showed a small effect size (*R*^2^ = 0.056), indicating a limited influence. One possible explanation for this outcome is that PMS symptoms exhibit significant individual differences and are influenced by complex and multifaceted characteristics, including emotional and psychological symptoms (Otsubo, 2018). Interoceptive sensory signals are transmitted to the brain via the ANS; however, this regulatory mechanism is disrupted by stress.

Since stress is one of the factors that exacerbate PMS, this study examined the effects of stress by comparing the mean values of various indices (subjective emotional scores, HCT scores, and ANS indices) between the PMS and the without PMS groups. The stress-free phase was set as the baseline for this comparison. The results for subjective emotional states showed that, as expected, a main effect of group was observed for subjective negative emotions and subjective stress scores. While the PMS group showed higher scores, no significant differences were observed between the groups in terms of subjective positive emotions.

Regarding the main effect of time, significant changes were observed in subjective positive effects, subjective negative effects, and subjective stress scores. These scores showed variation between the stress-free phase and immediately after stress, 30 min post-stress, and 60 min post-stress. However, no significant interaction effects were observed. These results suggest that stress induction was conducted appropriately.

The results of the HCT showed that the PMS group scored higher than the without PMS group. Additionally, a main effect of time was observed. Specifically, in the without PMS group, scores tended to increase from the baseline compared to those immediately after stress and 60 min post-stress induction. Conversely, no significant interaction effects were found. The HCT is reported to improve one’s ability to accurately perceive their own heartbeat when repeated (Schillings et al., 2022). The average scores of the HCT in this study were higher compared to those reported in previous studies (0.291–0.611; Desmedt et al., 2020). These results suggest that the findings of the HCT in this study may include both repetition and ceiling effects resulting from repeated implementations of the task.

The results of ANS activity showed no significant main effects of group for any indices. A main effect of time was observed significantly for heart rate, RR interval, HF, RMSSD, and pNN50.

Regarding heart rate, both the PMS group and the without PMS group showed decreases but the timings differed. In the without PMS group, a gradual decrease was observed from the pre-stress phase, whereas in the PMS group, a decrease was observed from immediately after stress to 60 min post-stress. Similarly, mean RR increased in both groups. However, in the without PMS group, a gradual increase was observed from the pre-stress phase, while in the PMS group, a marked increase was observed at 60 min post-stress compared to the pre-stress and immediately-after-stress phases. The HRV analysis revealed that HF increased from the pre-stress phase to 60 min post-stress, and RMSSD and pNN50 showed substantial increases in the PMS group from immediately after stress to 60 min post-stress.

However, for LF/HF, neither the main effect of time nor the interaction was significant. Therefore, it is necessary to carefully examine the effects of stress induction because HRV indices are considered indices of neural modulation and should be interpreted as reflecting changes in tonic neural activity relative to the mean (La Rovere et al., 2020). Thus, the LF/HF results may suggest that both groups exhibited similar ANS reactivity to stress.

To examine the degree of ANS response (Meng et al., 2022), Δ values of indices that showed a main effect of time in the frequency domain analysis were used. Although no group differences were observed significantly, both the main effect of time and the interaction were significant. ΔHF(P3 − P1) was greater than ΔHF(P2 − P1), and this effect was observed in the PMS group.

The observation that HF significantly increased exclusively in the PMS group during the recovery phase, despite the absence of significant differences between the groups at baseline, cannot be straightforwardly interpreted as a mere increase in parasympathetic activity or relaxation effects. This is because no differences in HF were significantly observed between the PMS and without PMS groups at baseline, indicating that the ANS activity of both groups was relatively stable in the absence of stress.

Under the application of a stress load, sympathetic activity typically becomes dominant, leading to a state of physiological arousal. However, this response is regulated by the baroreflex (baroreceptor reflex), which increases parasympathetic (vagal) activity in response to elevated blood pressure, resulting in a decrease in heart rate and suppression of sympathetic activity, even at the ventricular level (La Rovere et al., 2020). Following the relief of stress, the ANS typically initiates a process to restore homeostasis.

In this study, however, excessive parasympathetic activation was observed in the PMS group. This may be attributed to an overreaction of the baroreflex or vagal reflex, leading to an increase in HF. The prolonged increase in HF, exhibiting rebound effects (Mezzacappa et al., 2001), may lead to chronic fatigue and discomfort, making recovery difficult. Therefore, the phenomenon of increasing HF during the recovery phase is considered to reflect ANS imbalance experienced by women in the PMS group (Matsumoto et al., 2007a, 2007b). Additionally, these findings suggest that stress serves as an exacerbating factor for PMS symptoms.

These results suggest that both groups exhibited similar ANS reactivity to stress. However, a rebound effect was observed exclusively in the PMS group compared to the without PMS group, indicating that the PMS group may have impaired autonomic regulation timing, which could manifest as a rebound phenomenon during the recovery process. Furthermore, the results suggest that the PMS group would require more time for long-term adjustment during the recovery phase of stress response regulation, highlighting the potential for increased physiological burden and risk associated with stress in women with PMS.

In conclusion, women with PMS in the late luteal phase experience more negative emotional states and perceive greater stress compared to those without PMS. In the evaluation of interoception, high scores on the HCT and low scores on the MAIA suggest that women with PMS demonstrate high interoceptive accuracy, but their awareness of internal states is low. This mismatch may result in strong emotional experiences driven by accurate bodily information, while a reduced awareness and trust in internal states makes it difficult to integrate and interpret bodily sensations and signals as emotions. Thus, they may find it difficult to accurately recognize their own emotions. Additionally, the HF rebound effect observed exclusively in the PMS group after stress indicates impaired autonomic regulation timing, which may increase physiological burden and stress-related risks. Consequently, this could hinder their ability to process emotions appropriately and may contribute to a tendency toward chronic stress.

This study has several limitations. First, as the sample size is limited, caution must be exercised in generalizing the results. Second, data collection relied on self-reported measures, which may introduce bias. Third, the comparison of mean values for P1, P2, and P3 was conducted using only samples with significant stationarity, which limited the sample size. Considering these points, participant recruitment should be conducted more broadly to ensure generalizability. The prevalence of PMS varies across age groups, and its symptoms are diverse. Furthermore, while this study focused on the late luteal phase and was conducted in a laboratory setting, it is important to examine PMS conditions and the effects of stress during other phases, particularly the late follicular phase. Comparing these findings with the results of this study would provide valuable insights into understanding long-term changes.

## Data Availability

The original contributions presented in the study are included in the article/Supplementary material, further inquiries can be directed to the corresponding author.
