# Supplementary figures and images for: Women with premenstrual syndrome exhibit high interoceptive accuracy, but low awareness, with parasympathetic rebound responses from stress
Source: Front Neurosci. 2025 Feb 17;19:1489225. doi: 10.3389/fnins.2025.1489225 (PMC11872929; doi:10.3389/fnins.2025.1489225)

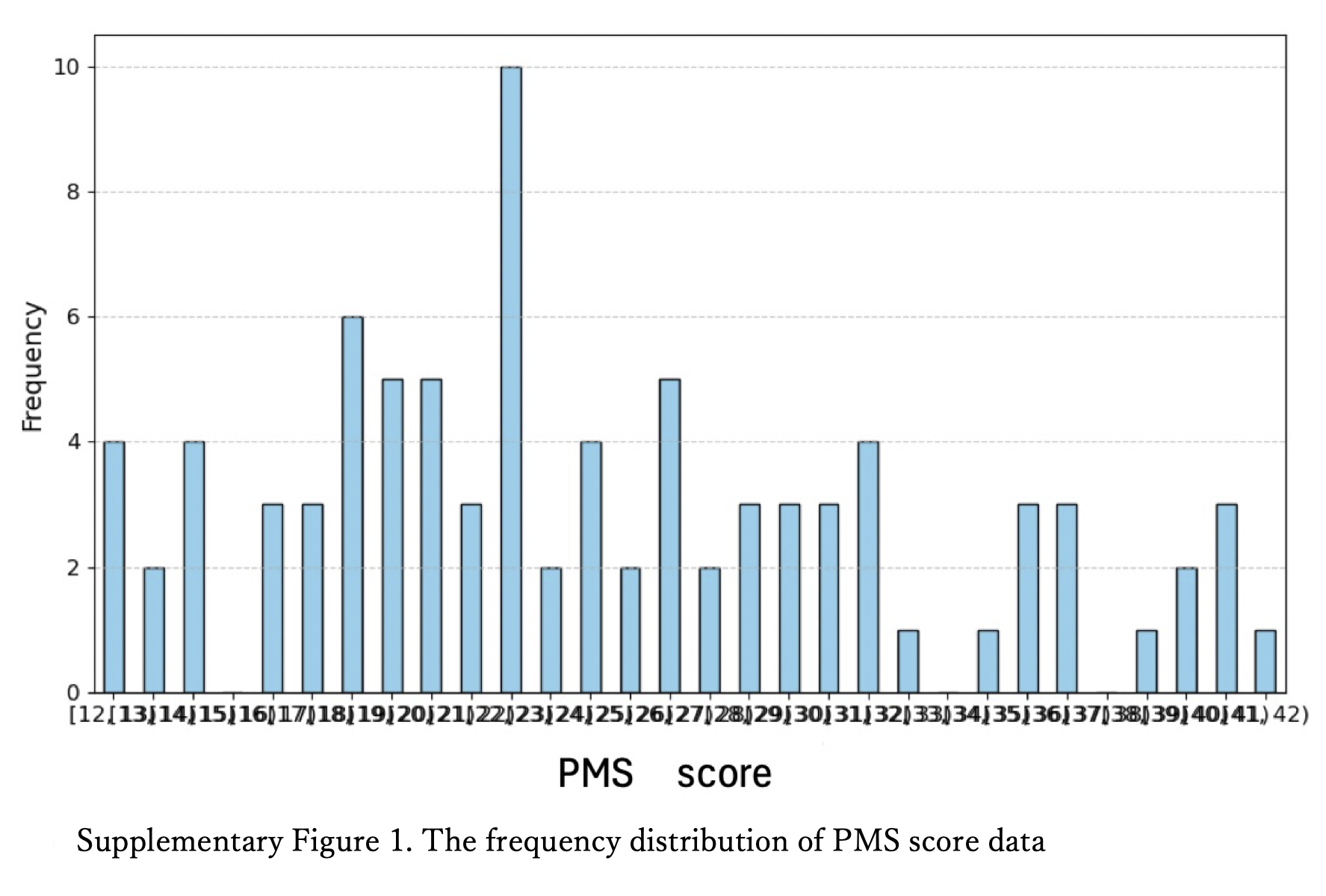

Supplement: Supplementary file 1 [file Image_1.TIFF]

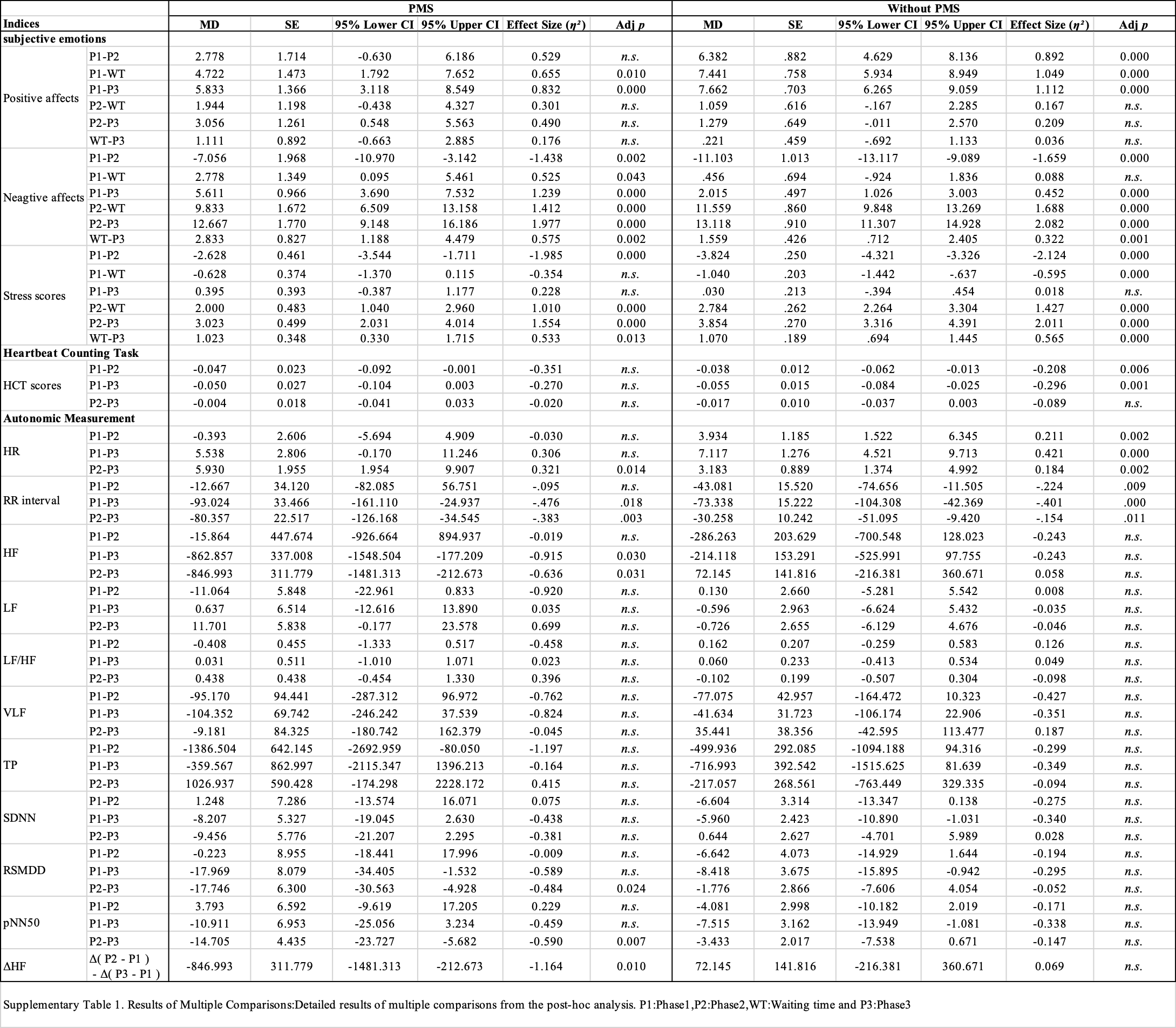

Supplement: Supplementary file 2 [file Image_2.TIFF]
